# Metabolic Flux Analysis of Lipid Biosynthesis in the Yeast *Yarrowia lipolytica* Using ^13^C-Labled Glucose and Gas Chromatography-Mass Spectrometry

**DOI:** 10.1371/journal.pone.0159187

**Published:** 2016-07-25

**Authors:** Huaiyuan Zhang, Chao Wu, Qingyu Wu, Junbiao Dai, Yuanda Song

**Affiliations:** 1 Colin Ratledge Center for Microbial Lipids, School of Agricultural Engineering and Food Science, Shandong University of Technology, Zibo, 255049, Shandong, People's Republic of China; 2 School of Food Science and Technology, Jiangnan University, Wuxi, 214122, Jiangsu, People’s Republic of China; 3 MOE Key Laboratory of Bioinformatics, School of Life Sciences, Tsinghua University, Beijing, 10084, People’s Republic of China; Nanyang Technological University, SINGAPORE

## Abstract

The oleaginous yeast *Yarrowia lipolytica* has considerable potential for producing single cell oil, which can be converted to biodiesel, a sustainable alternative to fossil fuels. However, extensive fundamental and engineering efforts must be carried out before commercialized production become cost-effective. Therefore, in this study, metabolic flux analysis of *Y*. *lipolytica* was performed using ^13^C-labeled glucose as a sole carbon source in nitrogen sufficient and insufficient media. The nitrogen limited medium inhibited cell growth while promoting lipid accumulation (from 8.7% of their biomass to 14.3%). Metabolic flux analysis showed that flux through the pentose phosphate pathway was not significantly regulated by nitrogen concentration, suggesting that NADPH generation is not the limiting factor for lipid accumulation in *Y*. *lipolytica*. Furthermore, metabolic flux through malic enzyme was undetectable, confirming its non-regulatory role in lipid accumulation in this yeast. Nitrogen limitation significantly increased flux through ATP:citrate lyase (ACL), implying that ACL plays a key role in providing acetyl-CoA for lipid accumulation in *Y*. *lipolytica*.

## Introduction

Due to recent petroleum crises and the worldwide accumulation of greenhouse gases in recent years, alternative fuels have attracted more and more interest. Biodiesel, which is produced via the esterification of vegetable oils or animal fats with lower alcohols, is an alternative to fossil fuel-derived diesel and is commercially available in Europe, the United States, and many other countries [1, 2]. The main drawback of this technology is that vegetable oils and animal fats are also consumed as food. As a alternative to plant oils and animal fats, oils produced by oleaginous microorganisms can also be used to produce biodiesel. Such oleaginous microorganisms have several advantages over current biodiesel production technologies, including high lipid yields and growth rates [3]. Almost all microbes are intrinsically capable of synthesizing fatty acids as cell membrane components and energy storage materials. However only a few microbes can accumulate intracellular lipid over 20% of their dry cell weight (DCW) and these microorganisms are regarded as oleaginous microorganisms [4].

The accumulation of high concentrations of lipids in oleaginous microbes is typically achieved by nutrition limitation in the culture medium, especially nitrogen limitation. When nitrogen is exhausted in the growth medium, the synthesis of intracellular protein and nucleotides ceases, and excess carbon sources are converted into lipids. These lipids are generally stored as neutral lipids such as diacylglycerols and triaclyglycerols [5]. It had been reported that lipid accumulation is initiated by a sharp decrease in adenosine monophosphate (AMP) concentrations. AMP is deaminized by AMP deaminase to produce inosine monophosphate (IMP) and ammonia, compensating for intracellular nitrogen depletion. In many oleaginous microorganisms, this deamination is triggered by nitrogen depletion from the growth medium. Consequently, the activity of mitochondrial isocitrate dehydrogenase (ICDH) in the tricarboxylic acid (TCA) cycle depends on intracellular AMP concentrations, ICDH activity decreases significantly in response to the decrease in AMP levels. This decrease in ICDH activity slows the TCA cycle, and the citrate accumulated within mitochondria is transported into the cytosol. In the cytosol, citrate is cleaved by ATP:citrate lyase (ACL) to generate oxaloacetate and acetyl-CoA. Acetyl-CoA is used for fatty acid biosynthesis and oxaloacetate can be converted to malate by cytoplasmic malate dehydrogenase (MDH). Cytosolic malate may be transported into mitochondria in exchange for citrate or may be decarboxylated to generate pyruvate and NADPH (which is used for lipid accumulation) by NADP^+^-dependent malic enzyme (NADP-ME) [6].

In the oleaginous yeast *Yarrowia lipolytica* the molecular mechanism that regulates lipid accumulation remains unclear. *Y*. *lipolytica* has considerable potential as a cell factory for oil production [7]. This microorganism can grow efficiently on several carbon sources (e.g., alkanes, fatty acids, ethanol, acetate, glucose, fructose and glycerol) and accumulate more than 40% lipid of their biomass. *Y*. *lipolytica* is considered a model organism for both laboratory study and industrial applications due to the availability of its genome sequence and the existence of genetic tools for gene manipulation [8–10]. Many studies on lipid accumulation in *Y*. *lipolytica* have demonstrated that lipid accumulation in this strain under nitrogen-limited condition is much more complex than in other oleaginous microorganisms. Our recent study demonstrated that the malic enzyme in *Y*. *lipolytica* does not exhibit NADP^+^-dependent activity and therefore, does not provide reducing power for fatty acid synthesis [11], although the malic enzyme plays a key role in lipid accumulation in *Mortierella alpina* and *Rhodotorula glutinis* [12,13]. According to metabolic flux analysis of engineered *Y*. *lipolytica* strain engineered to overexpress acetyl-CoA carboxylase (ACC) and diacylglycerol acyltransferase (DGAT), the pentose phosphate pathway was considered as primary NADPH provider for lipid accumulation [14]. The substrate for fatty acid biosynthesis, acetyl-CoA, is mainly supplied by ACL in this yeast, as shown by our recent study [15]. Overexpression of ACL from *Mus musculus* in *Y*. *lipolytica* led to a 2-fold increase in lipid accumulation [15], while deletion of ACL in *Y*. *lipolytica* markedly decreased lipid content [16, 17]. Other transcriptomic, metabolomic and lipidomic analyses of *Y*. *lipolytica* have also been performed, and these studies have broadened our understanding of the molecular and biochemical mechanisms underlying lipid accumulation in *Y*. *lipolytica* [18, 19]. However, the *in vivo* activities of central carbon metabolic pathways under nitrogen limited conditions are still far from clear.

Base on of ^13^C-labeling experiments, metabolic flux analysis has emerged as an integrated experimental and computational tool for identifying the biochemical networks and generating quantitative insight into the distribution of intracellular metabolic fluxes throughout central carbon metabolism [20]. Recently, metabolic flux analysis has been used to explore the biochemical mechanisms underlying lipid accumulation in oleaginous microorganisms. Xiong et al. [20] performed ^13^C-labeling experiments in the oleaginous microalga *Chlorella protothecoides*, and found that the malic enzyme was inactive *in vivo*, and under nitrogen limited culture condition the relative activity of pentose phosphate pathway was increased, reflecting an increased demand for NADPH during lipid accumulation. In the oleaginous yeast *Trichosporon cutaneum*, metabolic flux analysis demonstrated that the cytosolic malic enzyme is the primary source of NADPH and that ACL is the primary source of acetyl-CoA during lipid accumulation [21]. In this study, metabolic flux analysis based on ^13^C-labeled glucose was performed on *Y*. *lipolytica* grown under nitrogen-sufficient and nitrogen-limited growth condition.

## Materials and Methods

### Strain and culture condition

*Y*. *lipolytica* CICC1778 (ATCC 20460) used in this study was purchased from the China Center of Industrial Culture Collection (CICC). Intracellular carbon flux distributions were investigated in this strain grown in 5.0 g/liter ammonium sulfate (nitrogen-sufficient) or 0.5 g/liter ammonium sulfate (nitrogen-limited) supplemented with 10.0 g/liter glucose and 1.7 g/liter yeast nitrogen base without amino acids and ammonium sulfate (YNB).

Labeling experiments were performed in a 1 L shake flask with 100 mL of medium, which consisted of a 20%: 80% mixture of [U-^13^C] glucose (99%, Cambridge Isotope Laboratories): unlabeled glucose or 100% [1-^13^C] glucose (99%, Cambridge Isotope Laboratories). Cultures were incubated at 28°C and shaken at 200 rpm. Each culture was inoculated from a fresh preculture with a starting optical density (OD_600_) at 0.01. Each culture was performed in triplicate.

### Determination of physiological parameters

Cell growth during cultivation was monitored at OD_600_. Dry cell weight (DCW) was determined from 10 mL cell culture aliquots that were centrifuged for 10 min at 4°C and 10000 g, washed twice with cold distilled water, and dried at 110°C until their weights were constant.

Glucose concentrations in culture media were determined using a glucose oxidase test kit (Rongsheng Biotech., Shanghai, China) according to the manufacturer's instructions. The glucose oxidase kit has a lower detection limit of 0.4 mg/liter. Ammonium concentrations were measured using the indophenol method with ammonium sulfate as an ammonia standard. The indophenol method has a lower detection limit of 0.2 mg ammonium/liter. Five milliliters each of diluted solution A (10 g/liter phenol and 0.05 g/liter sodium nitroprusside) and diluted solution B (5 g/liter sodium hydroxide and 0.42 g/liter sodium hypochlorite) were mixed with 1 mL of sample. The absorbance of the solution was measured spectrophotometrically at 625 nm after incubation at 55°C for 3 min.

Protein contents were determined using the Lowry method [22]. Amino acid compositions were obtained using an L8800 amino acid analyzer (Hitachi Ltd.). Lipid contents and compositions were measured via gas chromatography as described below. The phenol-sulfuric acid method was used to determine intracellular carbohydrate and starch contents [23]. The KOH/UV method [24] and the modified Schneider method [25] were used to determine RNA and DNA concentrations, respectively. All experiments were performed in triplicate, and all data are reported as means ± standard deviations.

### Analysis of cell lipids and fatty acids

Yeast cells were collected via centrifugation and freeze-dried. Approximately 20 mg of freeze-dried cells were used for lipid content and fatty acid analysis. Pentadecanoic acid (15:0, Sigma) was added to samples of freeze-dried cells as an internal standard, and cell lipids were extracted with a chloroform/methanol (2:1, v/v) mixture. Extracted cell lipids were esterified with methanol, and the resulting fatty acid methyl esters (FAMEs) were analyzed via gas chromatography (GC-2010; Shimadzu Co., Kyoto, Japan) with a DB-Waxetr column (30 m by 0.32 mm; film thickness, 0.25 μm). The temperature program was as follows: 120°C for 3 min, ramp to 190°C at 5°C per min, ramp to 220°C at 4°C per min, and hold for 20 min. Each experiment was performed in triplicate, and all data are reported as means ± standard deviations.

### Proteinogenic amino acid preparation and GC-MS analysis

Proteinogenic amino acid preparation for GC-MS analysis followed standard protocols [26]. Briefly, mid-exponential phase cells (5 mL) were harvested by centrifugation, washed once with distilled water, and subsequently hydrolyzed in 6 M HCl at 110°C for 24 h. Hydrolysates were dried overnight at 80°C and dissolved in 125 μl of water-free pyridine. For GC-MS analysis, resuspended solutions were derivatized with 25 μl *N-tert*-butyldimethylsilyl-*N*-methyltrifuoroacetamide (TBDMS) at 80°C for 1 h. Samples were then analyzed via GC-MS. Each experiment was performed in triplicate, and all data are reported as means ± standard deviations.

As described in detail previously [20], GC-MS was performed using an Agilent GC-6890 gas chromatograph equipped with an Agilent HP-5MS column (30 m*0.25 mm*0.25 mm) directly connected to an MS-5975 mass spectrometer (Agilent). Helium was used as the carrier gas, and the column pressure was maintained at 8.21psi. The oven temperature was initially held at 60°C for 2 min, then increased to 180°C at a rate of 5°C min^-1^. The temperature was then raised to 260°C at a rate of 10°C min^-1^ and finally held at 260°C for 5 min. Other settings were as follows: 5 mL injection volume, 1:20 split ratio, and electron impact ionization of 70 eV.

### Metabolic modeling and flux analysis

As described in detail previously [20], for metabolic flux ratio analysis, a mass isotopomer distribution vector of each amino acid fragment MDV_α_ (Eq 1) was assigned according to the well-developed mathematic methodology [27],

$$MDV_{\alpha }^{*}=\left[\begin{array}{c} \left(m_{0}\right)\\ \left(m_{1}\right)\\ \vdots \\ \left(m_{n}\right) \end{array}\right]\sum_{i=0}^{n}m_{i}=1$$ (1)

Where m_0_ is the fractional abundance of fragments with monoisotopic masses and m_i>0_ is the abundance of molecular with higher masses.

To obtain the exclusive mass isotope distribution of the carbon skeleton MDV_α_*, the GC-MS data were corrected for the natural isotope abundances of oxygen (O), nitrogen (N), hydrogen (H), silicon (Si), sulfur (S) and carbon (C) atoms in the amino acids using a correction matrix (Eq 2)

$$MDV_{\alpha }^{*}=C_{corr,CHONSSi}\times MDV_{\alpha }$$ (2)

The correction matrix C_corr,CHONSSi_ was obtained from the correction matrices for all individual atom species (Eq 3)

$$C_{corr,CHONSSi}=C_{corr,C}\times C_{corr,H}\times C_{corr,O}\times C_{corr,N}\times C_{corr,S}\times C_{corr,Si}$$ (3)

And then the contribution of ^13^C from the unlabeled biomass in culture inocula was subtracted from MDV_α_*, yielding mass distribution vector of amino acid MDV_AA_ according to Eq 4.

$$MDA_{AA}=\frac{MDV_{\alpha }^{*}-f_{unlabeled}\cdot MDV_{unlabeled,n}}{1-f_{unlabeled}}$$ (4)

Where *f*_unlabeled_ is the fraction of the unlabeled biomass, which was caused by inoculation and MDV_unlabled,*n*_ is the mass distribution of an unlabeled fragment with *n* C atoms.

### Metran software for ^13^C labeled metabolic flux analysis

In this study, metabolic fluxes and confidence intervals were determined via the simultaneous fitting of external fluxes and the mass isotopomer abundances of intracellular amino acids to a detailed metabolic network model of yeast cells using Metran. Metran is a software program developed by Maciek Antoniewicz for ^13^C labeled metabolic flux analysis, tracer experiment design and statistical analysis [28].

### Statistical analysis

In this study, each experiment was performed in triplicate. All data are reported as means ± standard deviations and were analyzed with ANOVA and a t-test.

## Results

### Fermentation profiles and the establishment of a metabolic steady state

**Fig 1:**
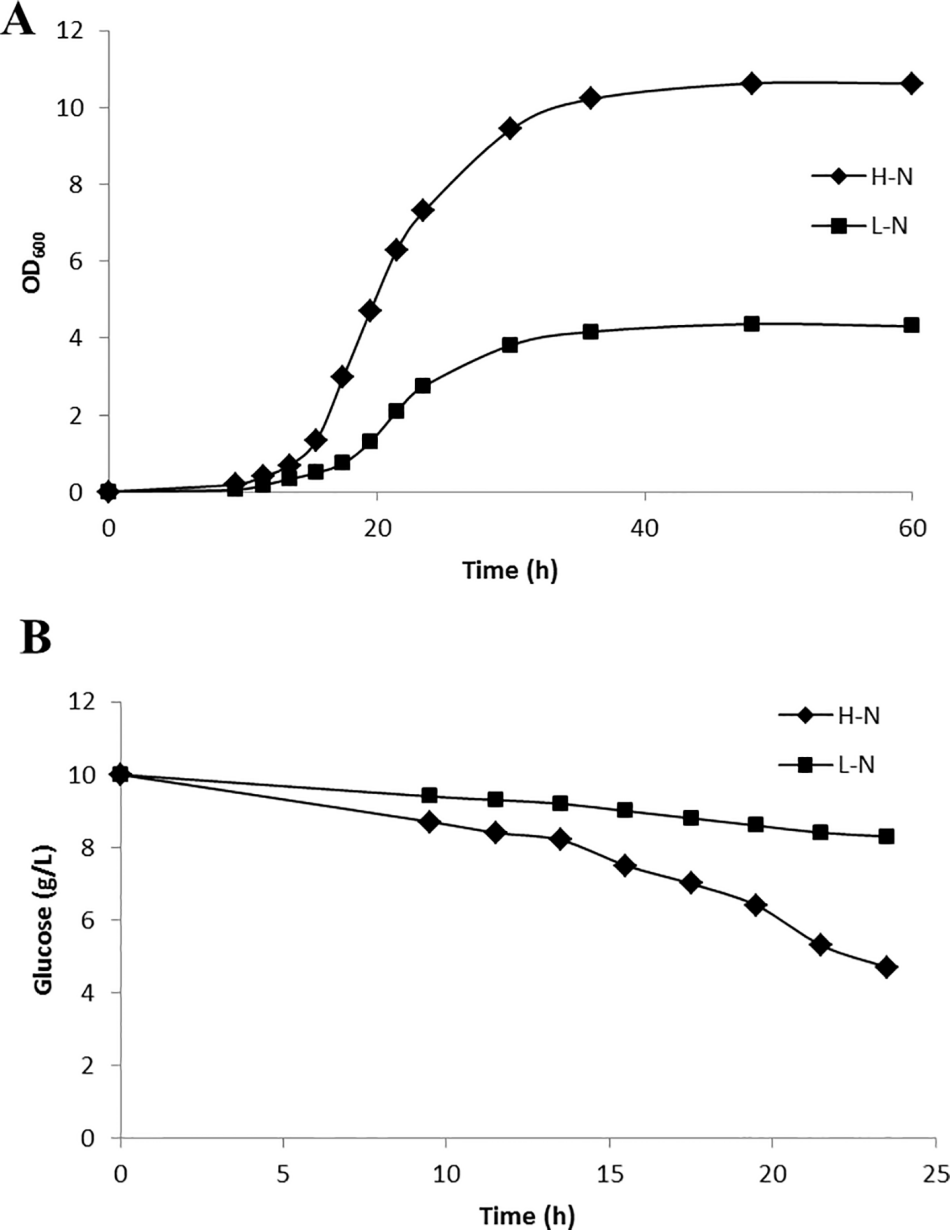
Growth curve (A) and residual glucose concentration (B) for *Y*. *lipolytica* cultivated in high nitrogen medium (H-N) and low nitrogen medium (L-N) with glucose as a sole carbon source. Each experiment was performed in triplicate, and all data are reported as means ± standard deviations.

*Y*. *lipolytica* was cultivated in high nitrogen and low nitrogen media with glucose as the sole carbon source. This strain exhibited similar growth profiles in both culture conditions. Following inoculation, *Y*. *lipolytica* exhibited a 12 h lag phase for 12 h and an exponential phase from 12 h to 30 h in both culture conditions. However, the final cell concentrations (OD_600_) were different. The OD_600_ of the yeast culture grown in high nitrogen medium was approximately 11, while the OD_600_ of the culture grown in low nitrogen medium was approximately 4 (Fig 1A). Not surprisingly, nitrogen limitation resulted in a cell growth rate (0.168 h^-1^) lower than that obtained in the high nitrogen medium (Table 1). Glucose consumption profiles also differed depending on nitrogen concentration: the strain consumed glucose faster in the high nitrogen medium (2.900 mmol g^-1^ h^-1^) than in the low nitrogen medium (1.017 mmol g^-1^ h^-1^) (Fig 1B). Nitrogen limitation therefore resulted in a decreased glucose uptake rate.

**Table 1:** Growth characteristics of *Y*. *lipolytica* in high nitrogen medium (H-N) and low nitrogen medium (L-N). μ, specific cell growth rate; q_glc,_ specific glucose consumption rate.

	μ (h^-1^)	q_glc_ (mmolg^-1^h^-1^)
H-N	0.190	2.900
L-N	0.168	1.017

*Y*. *lipolytica* was cultured in a 20%:80% mixture of [U-^13^C] glucose: unlabeled glucose or 100% [1-^13^C] glucose to facilitate ^13^C-labeled metabolic flux analysis. Metabolic and isotopic steady states are prerequisites for ^13^C-labeled metabolic flux analyses. These prerequisites mean that all intermediate concentrations and fluxes remain constant throughout the ^13^C-labeled experiment. In batch cultures, metabolic and isotopic steady states exist during exponential growth, where growth rates and isotopomer fractions are constant. Therefore, 20 h was chosen as the time point for biomass composition analysis and ^13^C-labeled flux experiments.

### Influence of nitrogen concentration on biomass composition

**Fig 2:**
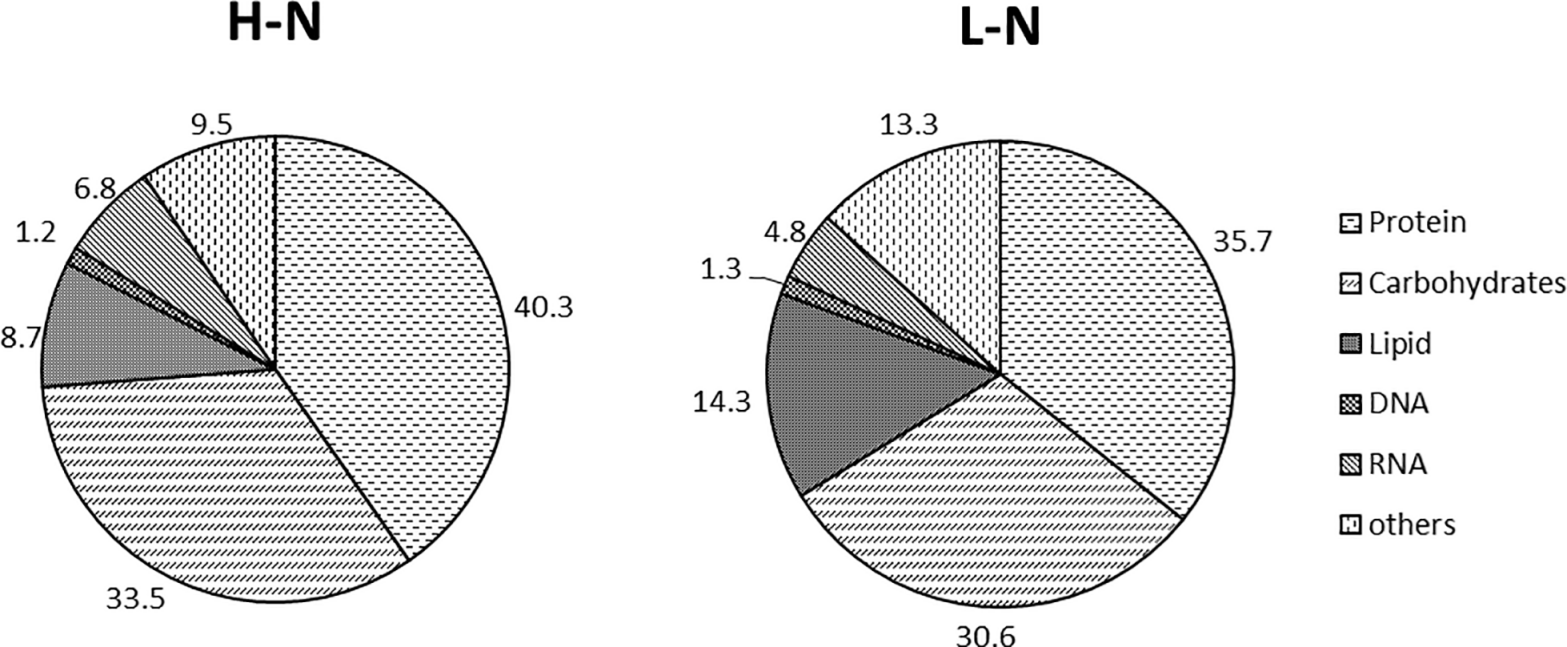
Structural units for biomass formation in *Y*. *lipolytica* cultivated in high nitrogen medium (H-N) and low nitrogen medium (L-N). Numbers represent the percentages of corresponding biomass components (g g^-1^ dry cell weight). Each experiment was performed in triplicate, and all data are reported as means ± standard deviations.

To calculate absolute fluxes, the amount of metabolites withdrawn to fulfill biosynthetic demands must be known. First, the macromolecular composition of *Y*. *lipolytica* was analyzed (Fig 2). This macromolecular composition encompasses the five major cell components (carbohydrates, lipids, proteins, DNA and RNA), which account for 85% of the total dry cell weight. Nitrogen concentration did not significantly influence on DNA or RNA levels. In contrast, lipid, protein and carbohydrate contents differed dramatically between the high and low nitrogen media. The lipid content of *Y*. *lipolytica* cultivated in low nitrogen medium was 14.3% of dry cell weight, nearly 2-fold higher than the lipid content of *Y*. *lipolytica* cultivated in high nitrogen medium (8.7%). Second, the macromolecular composition of *Y*. *lipolytica* was also measured for the prediction of intracellular fluxes. This macromolecular composition consists of the compositions of proteinogenic amino acids, fatty acids, DNA and RNA. The nucleotide composition of the DNA was obtained from the genome sequence, and the same composition was assumed for RNA. The DNA and RNA compositions calculated from the genome sequence of *Y*. *lipolytica* are shown in Table 2. The contents of nearly all proteinogenic amino acids were similar between cultures grown under nitrogen-sufficient and nitrogen-limited conditions. Only the contents of lysine, methionine and serine differed between the two culture conditions (Table 3). Nitrogen limitation did not affect saturated fatty acid (palmitic and stearic acids) biosynthesis in yeast cells. However, nitrogen limitation increased the content of monounsaturated fatty acids (palmitoleic and oleic acids) and decreased the content of linoleic acid (S1 Fig).

**Table 2:** Nucleotide compositions of DNA and RNA in *Y*. *lipolytica* cultivated in H-N and L-N medium.

DNA (RNA) composition(%)	H-N	L-N
A	25.5	25.5
T(U)	25.5	25.5
G	24.5	24.5
C	24.5	24.5

**Table 3:** Amino acid composition in *Y*. *lipolytica* cultivated in high nitrogen medium (H-N) and low nitrogen medium (L-N).

Amino acid composition(%)	H-N	L-N
Ala	11.5 ± 0.6	12.6 ± 0.4
Arg	5.0 ± 0.3	5.3 ± 0.4
Asn	5.6 ± 0.9	5.6 ± 1.4
Asp	5.6 ± 0.9	5.6 ± 1.4
Cys	0.1 ± 0.0	0.1 ± 0.0
Gln	8.1± 1.1	7.7 ± 1.1
Glu	8.1± 1.1	7.7 ± 1.1
Gly	8.7 ± 0.4	8.8 ± 0.9
His	1.9 ± 0.2	1.8 ± 0.1
Ile	2.7 ± 0.2	2.9 ± 0.2
Leu	5.8 ± 0.3	6.1 ± 0.3
Lys	9.2 ± 1.1	7.6 ± 0.5
Met	1.5 ± 0.1	0.7 ± 0.1
Phe	3.1 ± 0.4	3.3 ± 0.4
Pro	5.4 ± 0.5	5.2 ± 0.4
Ser	6.8 ± 0.2	7.6 ± 0.4
Thr	5.7 ± 0.2	6.0 ± 0.7
Trp	0.1 ± 0.0	0.1 ± 0.0
Tyr	1.7 ± 0.1	1.9 ± 0.1
Val	4.6 ± 0.1	4.8 ± 0.1

### *Y*. *lipolytica* metabolic network construction of and metabolic flux analysis

A model of the metabolic networks to be studied is a prerequisite for ^13^C-labeled metabolic flux analysis method. For this study, a model of the central carbon metabolism network is required for flux analysis. This network was constructed based on the genome sequence of *Y*. *lipolytica*. The genomic information contained within the sequence database was organized into various essential pathways, including the glycolysis/gluconeogenesis, the pentose phosphate pathway, the TCA cycle, fatty acid biosynthesis, amino acid metabolic pathways and nucleotide metabolism. Metabolic pathway information for *Y*. *lipolytica* was also available from previous studies. This information was used to identify the metabolic networks in *Y*. *lipolytica*. All biochemical reactions are listed in the Supporting Information.

To calculate metabolic fluxes, the abundances of ^13^C-labeled proteinogenic amino acid carbon skeletons must be determined via GC-MS or Nuclear Magnetic Resonance (NMR). In recent years, GC-MS has become a sensitive and useful tool for the estimation of ^13^C-labeled fluxes. To track the mass isotopomer patterns of proteinogenic amino acids in *Y*. *lipolytica*, cells were harvested from mid-exponential growth phase cultures grown with ^13^C-labeled glucose under nitrogen-rich and nitrogen-limited conditions. Samples were then subjected to hydrolysis and derivatization for GC-MS analysis. Because ^13^C-labeled metabolic flux analysis requires exclusive mass distributions of carbon skeletons, raw amino acid fragment mass data were corrected for natural isotope abundances (Table 4). Because a mixture of 20% [U- ^13^C] glucose and 80% unlabeled glucose was used as the substrate, all metabolites were expected to have fractional labeling (FL) values of approximately 0.2. FL values for proteinogenic amino acid fragments were close to this theoretical FL (S1 Table). Based on GC-MS measurements and amino acid biosynthesis schemes, corrected amino acid fragment abundance data were used to calculate the distribution of fluxes in central carbon metabolism.

**Table 4:** Mass isotopomer distribution of TBDMS-derivatized protein-bound amino acids (corrected) The symbols of fragments denote the cracking patterns of TBDMS-derivatized amino acids: (M-15) ^+^, (M-57) ^+^, (M-85) ^+^, (M-159) ^+^, f302, and side chain (sc) fragments.

Fragments	High nitrogen condition										Low nitrogen condition									
	m	m+1	m+2	m+3	m+4	m+5	m+6	m+7	m+8	m+9	m	m+1	m+2	m+3	m+4	m+5	m+6	m+7	m+8	m+9	
M_ala_057	0.7019	0.0766	0.0464	0.1679	0.0000	0.0000	0.0000	0.0000	0.0000	0.0000	0.7001	0.0899	0.0515	0.1584	0.0000	0.0000	0.0000	0.0000	0.0000	0.0000	
M_ala_085	0.7462	0.0522	0.2016	0.0000	0.0000	0.0000	0.0000	0.0000	0.0000	0.0000	0.7409	0.0664	0.1928	0.0000	0.0000	0.0000	0.0000	0.0000	0.0000	0.0000	
M_asx_057	0.5178	0.2367	0.1377	0.0850	0.0218	0.0000	0.0000	0.0000	0.0000	0.0000	0.5488	0.2151	0.1430	0.0815	0.0116	0.0000	0.0000	0.0000	0.0000	0.0000	
M_asx_085	0.6022	0.2412	0.1329	0.0232	0.0000	0.0000	0.0000	0.0000	0.0000	0.0000	0.5835	0.2431	0.1410	0.0323	0.0000	0.0000	0.0000	0.0000	0.0000	0.0000	
M_asx_302	0.6895	0.1781	0.1324	0.0000	0.0000	0.0000	0.0000	0.0000	0.0000	0.0000	0.7444	0.1325	0.1230	0.0000	0.0000	0.0000	0.0000	0.0000	0.0000	0.0000	
M_glx_057	0.4554	0.2083	0.2451	0.0533	0.0269	0.0110	0.0000	0.0000	0.0000	0.0000	0.4622	0.2082	0.2496	0.0282	0.0434	0.0084	0.0000	0.0000	0.0000	0.0000	
M_glx_085	0.5050	0.2403	0.2092	0.0157	0.0299	0.0000	0.0000	0.0000	0.0000	0.0000	0.5090	0.2177	0.2455	0.0011	0.0266	0.0000	0.0000	0.0000	0.0000	0.0000	
M_glx_302	0.7028	0.2380	0.0412	0.0000	0.0000	0.0000	0.0000	0.0000	0.0000	0.0000	0.7269	0.1461	0.1269	0.0000	0.0000	0.0000	0.0000	0.0000	0.0000	0.0000	
M_gly_057	0.7030	0.1207	0.1763	0.0000	0.0000	0.0000	0.0000	0.0000	0.0000	0.0000	0.6954	0.1366	0.1680	0.0000	0.0000	0.0000	0.0000	0.0000	0.0000	0.0000	
M_gly_085	0.7673	0.2327	0.0000	0.0000	0.0000	0.0000	0.0000	0.0000	0.0000	0.0000	0.7511	0.2489	0.0000	0.0000	0.0000	0.0000	0.0000	0.0000	0.0000	0.0000	
M_ile_015	0.4573	0.1462	0.2310	0.0933	0.0419	0.0223	0.0080	0.0000	0.0000	0.0000	0.4715	0.1369	0.2683	0.0563	0.0526	0.0116	0.0029	0.0000	0.0000	0.0000	
M_ile_085	0.4626	0.1976	0.2224	0.0813	0.0283	0.0078	0.0000	0.0000	0.0000	0.0000	0.4743	0.1985	0.2248	0.0608	0.0329	0.0087	0.0000	0.0000	0.0000	0.0000	
M_leu_015	0.4344	0.1093	0.3459	0.0171	0.0802	0.0069	0.0062	0.0000	0.0000	0.0000	0.4796	0.1337	0.2879	0.0279	0.0617	0.0048	0.0043	0.0000	0.0000	0.0000	
M_leu_085	0.4680	0.1903	0.2411	0.0587	0.0337	0.0081	0.0000	0.0000	0.0000	0.0000	0.4928	0.1816	0.2333	0.0541	0.0299	0.0084	0.0000	0.0000	0.0000	0.0000	
M_phe_057	0.4113	0.1071	0.1298	0.1586	0.0920	0.0447	0.0296	0.0164	0.0058	0.0046	0.4102	0.1073	0.1413	0.1525	0.0861	0.0437	0.0328	0.0196	0.0022	0.0042	
M_phe_085	0.3838	0.1192	0.2306	0.0536	0.1253	0.0313	0.0426	0.0073	0.0063	0.0000	0.4272	0.1093	0.1944	0.0664	0.1052	0.0419	0.0362	0.0136	0.0059	0.0000	
M_phe_302	0.7550	0.0718	0.1733	0.0000	0.0000	0.0000	0.0000	0.0000	0.0000	0.0000	0.7782	0.0478	0.1740	0.0000	0.0000	0.0000	0.0000	0.0000	0.0000	0.0000	
M_phe_sc	0.4076	0.1745	0.1359	0.0825	0.0999	0.0387	0.0379	0.0229	0.0000	0.0000	0.4166	0.1757	0.1390	0.0816	0.0995	0.0376	0.0359	0.0141	0.0000	0.0000	
M_pro_057	0.4327	0.2224	0.2202	0.0808	0.0331	0.0108	0.0000	0.0000	0.0000	0.0000	0.4793	0.2244	0.2057	0.0606	0.0212	0.0089	0.0000	0.0000	0.0000	0.0000	
M_pro_085	0.5107	0.2206	0.2085	0.0367	0.0235	0.0000	0.0000	0.0000	0.0000	0.0000	0.5132	0.2463	0.1748	0.0479	0.0179	0.0000	0.0000	0.0000	0.0000	0.0000	
M_ser_057	0.7062	0.0938	0.0627	0.1374	0.0000	0.0000	0.0000	0.0000	0.0000	0.0000	0.7002	0.1262	0.0323	0.1413	0.0000	0.0000	0.0000	0.0000	0.0000	0.0000	
M_ser_085	0.6991	0.1295	0.1714	0.0000	0.0000	0.0000	0.0000	0.0000	0.0000	0.0000	0.7353	0.1220	0.1426	0.0000	0.0000	0.0000	0.0000	0.0000	0.0000	0.0000	
M_ser_302	0.6842	0.1447	0.1711	0.0000	0.0000	0.0000	0.0000	0.0000	0.0000	0.0000	0.7021	0.1266	0.1713	0.0000	0.0000	0.0000	0.0000	0.0000	0.0000	0.0000	
M_thr_057	0.5864	0.1698	0.1304	0.0936	0.0198	0.0000	0.0000	0.0000	0.0000	0.0000	0.5382	0.2264	0.1674	0.0362	0.0318	0.0000	0.0000	0.0000	0.0000	0.0000	
M_thr_085	0.6196	0.2316	0.1011	0.0477	0.0000	0.0000	0.0000	0.0000	0.0000	0.0000	0.6100	0.2089	0.1119	0.0691	0.0000	0.0000	0.0000	0.0000	0.0000	0.0000	
M_thr_sc	0.7400	0.1069	0.1531	0.0000	0.0000	0.0000	0.0000	0.0000	0.0000	0.0000	0.7066	0.1366	0.1567	0.0000	0.0000	0.0000	0.0000	0.0000	0.0000	0.0000	
M_val_057	0.5400	0.1024	0.1806	0.1241	0.0209	0.0322	0.0000	0.0000	0.0000	0.0000	0.5422	0.1119	0.1754	0.1282	0.0104	0.0320	0.0000	0.0000	0.0000	0.0000	
M_val_085	0.5492	0.0946	0.3061	0.0115	0.0387	0.0000	0.0000	0.0000	0.0000	0.0000	0.5554	0.1125	0.2884	0.0095	0.0342	0.0000	0.0000	0.0000	0.0000	0.0000	
M_val_302	0.6939	0.1073	0.1988	0.0000	0.0000	0.0000	0.0000	0.0000	0.0000	0.0000	0.7190	0.0815	0.1995	0.0000	0.0000	0.0000	0.0000	0.0000	0.0000	0.0000	
M_tyr_057	0.3879	0.1108	0.1632	0.1141	0.1105	0.0348	0.0481	0.0194	0.0056	0.0057	0.4056	0.1114	0.1208	0.1510	0.0956	0.0448	0.0393	0.0210	0.0011	0.0093	
M_tyr_085	0.4159	0.1057	0.2223	0.0647	0.1099	0.0323	0.0384	0.0025	0.0085	0.0000	0.4214	0.1043	0.2080	0.0768	0.1186	0.0132	0.0469	0.0083	0.0025	0.0000	
M_tyr_302	0.7719	0.0563	0.1718	0.0000	0.0000	0.0000	0.0000	0.0000	0.0000	0.0000	0.7707	0.0709	0.1584	0.0000	0.0000	0.0000	0.0000	0.0000	0.0000	0.0000	

**Fig 3:**
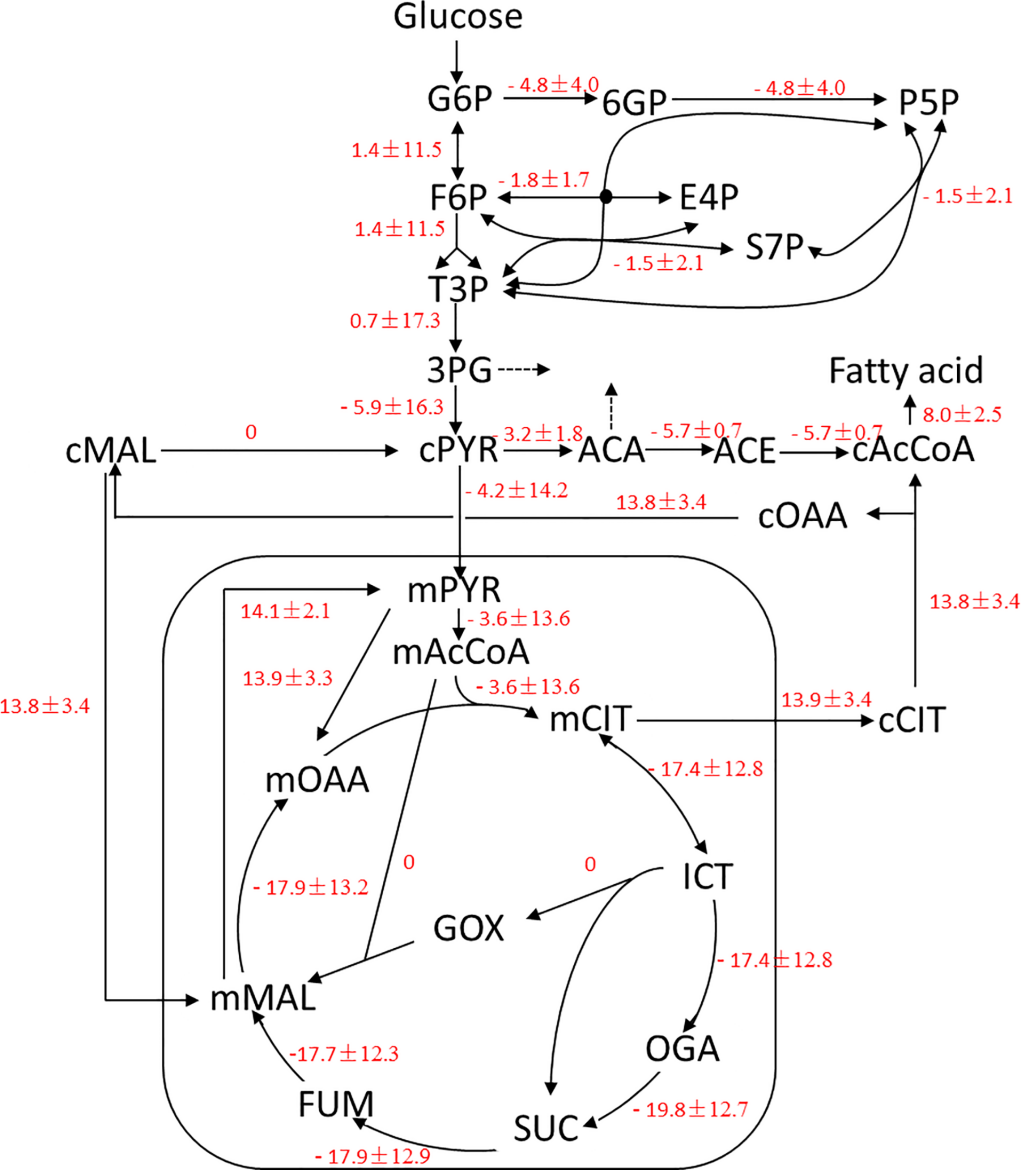
Differences in metabolic fluxes between the high and low nitrogen culture conditions. Data are shown as the delta flux value (LN—HN) for the low and high nitrogen culture conditions. Results were obtained from three replicate experiments. AcCoA, acetyl-coenzymeA; CIT, citrate; E4P, erythrose-4- phosphate; F6P, fructose-6-phosphate; FBP, fructose-1,6-bisphosphate; FUM, fumarate; G3P, glucose-3-phosphate; G6P, glucose-6-phosphate; GOX, glyoxylate; ICT, isocitrate; MAL, malate; OAA, oxaloacetate; OGA, α-ketoglutarate; 3PG, 3-phosphoglycerate; P5P, pentose-5-phosphate; 6PG, 6-phosphogluconate; PYR, pyruvate; Ru5P, ribulose-1,5-bisphosphate; S7P, sedoheptulose-7-phosphate; SUC, succinate.; T3P, triose-3-phosphate; m, mitochondrial; c, cytoplasmic.

Metabolic flux ratio analysis [27] was used to directly identify active metabolic networks in *Y*. *lipolytica*. Flux distributions inferred from the labeling results are summarized in S2 Fig, and differences in the metabolic flux between the two cultivation conditions are shown in Fig 3. The glyoxylate bypass, which redirects flux in the TCA cycle from isocitrate to malate, serves an anaplerotic function for cell growth on acetate or fatty acids and replenishes the carbon skeletons withdrawn from the TCA cycle for biosynthesis. As shown in Fig 3, flux through the glyoxylate bypass was not observed when *Y*. *lipolytica* was grown on glucose. Malic enzyme, which is considered the main provider of NADPH for fatty acid biosynthesis in oleaginous microorganisms, was also inactive in *Y*. *lipolytica*. In addition, other NADPH-generating biochemical reactions were slightly inhibited by nitrogen limitation. The pentose phosphate pathway was not regulated by the nitrogen concentration of the medium. However, isocitrate dehydrogenase activity was 20% lower in the low nitrogen medium compared to the high nitrogen medium. Of the metabolic pathways investigated, ACL, which converts citrate to acetyl-CoA for fatty acid biosynthesis, was most affected by the nitrogen concentration of the medium. ACL was inactive in *Y*. *lipolytica* grown in nitrogen-rich medium but was highly upregulated in *Y*. *lipolytica* grown in nitrogen-limited medium (Fig 3). The differences inmetabolic flux between nitrogen-rich medium and nitrogen-limited medium are shown in S2 Fig.

## Discussion

Malic enzyme has been suggested as the primary NADPH producer for lipid accumulation in oleaginous microorganisms [29], however, it is not the case in the yeast *Y*. *lipolytica* [11]. According to the flux data generated in this study, the NADP^+^-dependent activity of malic enzyme was inactive in *Y*. *lipolytica* regardless of the nitrogen concentration in the growth media. A similar result was observed for malic enzyme in the microalgae *C*. *protothecoides* [20]. Genomic analysis indicates that a single malic enzyme gene is present in the *Y*. *lipolyitca* genome [30] and predicts that the enzyme is located in the mitochondria. In addition, an *in vitro* activity study demonstrated that the *Y*. *lipolyitca* malic enzyme prefers NAD^+^ over NADP^+^ [11]. Previously, overexpression of an endogenous malic enzyme or an NADP^+^-dependent malic enzyme from the oleaginous fungus *M*. *alpina* did not alter lipid accumulation in *Y*. *lipolytica* [11]. Taken together, these results suggested that malic enzyme does not play an important role in providing the NADPH required for fatty acid biosynthesis in *Y*. *lipolytica*.

Apart from malic enzyme, other major NADPH generating enzymes that are involved in lipid biosynthesis include glucose-6-phosphate dehydrogenase and 6-phosphogluconate dehydrogenase from the pentose phosphate pathway. In some cases, cytoplasmic NADP^+^-dependent isocitrate dehydrogenase is also involved in lipid biosynthesis [31]. The involvement of these enzymes in lipid accumulation in oleaginous microorganisms was recently investigated [29]. In this study, the NADP^+^-dependent malic enzyme was found to be inactive, suggesting that the pentose phosphate pathway is the major provider of NADPH *Y*. *lipolytica*. However, flux through the pentose phosphate pathway is slightly downregulated under nitrogen-limited conditions. This apparent discrepancy may be explained by the fact that *Y*. *lipolytica* accumulated only 14% lipids (w/w, CDW) and exhibited decreased protein content under nitrogen-limited conditions, indicating that the pentose phosphate pathway supplies sufficient NADPH for lipid biosynthesis. Excess NADPH generated under nitrogen-rich conditions could be used by the strain to increase higher protein content and to synthesize other metabolites. Indeed, overexpression of glucose-6-phosphate dehydrogenase or 6-phosphogluconate dehydrogenase did not significantly increase lipid accumulation in this yeast (unpublished data). These results suggest that the pentose phosphate pathway produces sufficient NADPH for lipid biosynthesis in this low-oleaginous wild type strain and does not limit lipid accumulation. In a recent report, ^13^C-labeled metabolic flux analysis showed that in a high-oleaginous yeast strain engineered to overexpress ACC and DGAT, pentose phosphate pathway activity was upregulated compared to a the control strain. This result demonstrates that the pentose phosphate pathway is the primary source of NADPH for lipid accumulation in *Y*. *lipolytica* [14].

The two carbon unit acetyl-CoA molecule, which is primarily generated by the TCA cycle, is the building block for fatty acid biosynthesis. In nitrogen-limited condition, the flux through the TCA cycle in *Y*. *lipolytica* was approximately 20% lower than compared to in the nitrogen-rich conditions. This finding is consistent with early biochemical studies on lipid accumulation in the oleaginous fungi *M*. *alpina* and *Mucor circinelloides*, which found that nitrogen limitation leads to a decrease in the activity of TCA cycle enzymes and to the accumulation of cytosolic citrate. This cytosolic citrate is then converted to acetyl-CoA by ACL for fatty acid synthesis [31]. We found that the flux of carbon through ACL for acetyl-CoA production was increased under nitrogen-limited conditions. Consistently, ^13^C-labeled metabolic flux analysis has indicated that ACL is the primary source for excess lipid accumulation in the oleaginous yeast *T*. *cutaneum* [21]. In addition, previous work by our group demonstrated that heterogeneous expression of a *Mus musculus* ACL, which has a low K_m_ (0.05 mM) for citrate, increased lipid production in *Y*. *lipolytica* by 200% [15]. A 30% knockdown of endogenous ACL activity correspondingly decreased lipid production by 36% in *Y*. *lipolytica* [17]. Therefore, ACL plays a vital role in lipid accumulation in the oleaginous yeast *Y*. *lipolytica*.

In conclusion, metabolic flux analysis using ^13^C-labeled glucose as a sole carbon source was performed on *Y*. *lipolytica* grown in nitrogen-rich and nitrogen-limited mediums. Nitrogen limitation led to increased lipid accumulation. The results of metabolic flux analysis showed that malic enzyme is inactivity in *Y*. *lipolytica*, indicating that the enzyme does not regulate lipid accumulation in this yeast strain. Furthermore, nitrogen concentration in the medium was found to have little influence on the metabolic flux through the pentose phosphate pathway, suggesting that the pentose phosphate pathway yields sufficient NADPH for lipid biosynthesis and is not a limiting factor for lipid accumulation. Meanwhile, nitrogen limitation significantly increased flux through ACL, indicating that this enzyme plays a key role in providing acetyl-CoA for lipid accumulation in *Y*. *lipolytica*. Taken together, these results provide some insights into the roles of key enzymes for lipid accumulation in *Y*. *lipolytica*.

## Supporting Information

S1 Fig Fatty acid composition of *Y*. *lipolytica* cultivated in high nitrogen medium (H-N) and a low nitrogen medium (L-N). Each experiment was performed in triplicate, and all data are reported as means ± standard deviations.
(TIF)

S2 Fig Metabolic flux distribution of *Y*. *lipolytica* cultivated in high nitrogen medium (A) and low nitrogen medium (B). A value of 0 in the metabolic flux map indicates a very low flux (below 0.5).
(TIF)

S1 File Biochemical reactions consisting the metabolic networks of *Y*. *lipolytica* (m, mitochondrial; c, cytoplasmic). (DOCX)

S1 Table ^13^C fractional labeling (FL) of proteinogenic amino acids fragments from high nitrogen cultivation (H-N) and low nitrogen cultivation (L-N). The theoretical FL value is 0.2, equal to the labeling content of the input glucose.
(DOCX)
