# Supplementary figures and images for: Metabolic Flux Analysis of Lipid Biosynthesis in the Yeast Yarrowia lipolytica Using 13C-Labled Glucose and Gas Chromatography-Mass Spectrometry
Source: PLoS One. 2016 Jul 25;11(7):e0159187. doi: 10.1371/journal.pone.0159187 (PMC4959685; doi:10.1371/journal.pone.0159187)

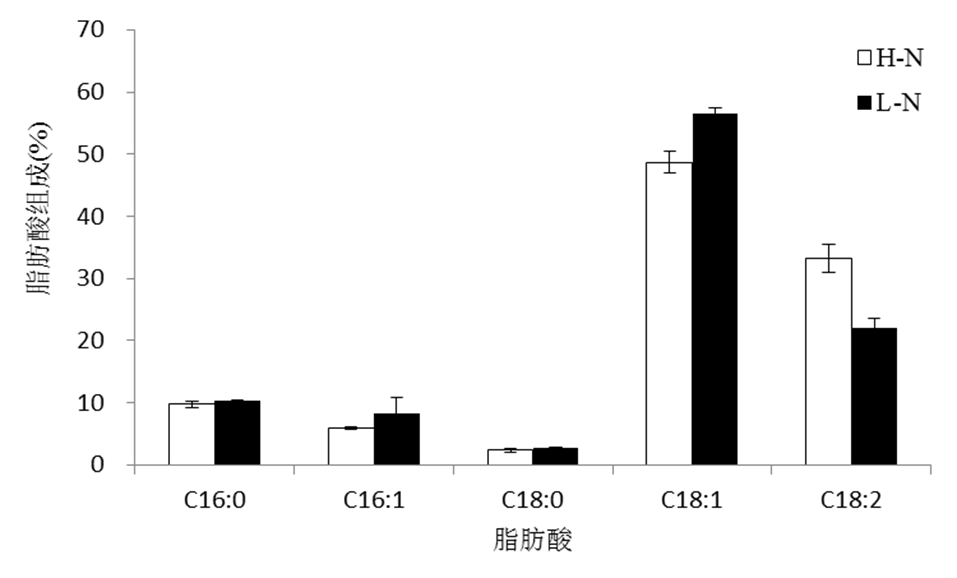

Supplement: S1 Fig — Each experiment was performed in triplicate, and all data are reported as means ± standard deviations. (TIF) [file pone.0159187.s001.tif]

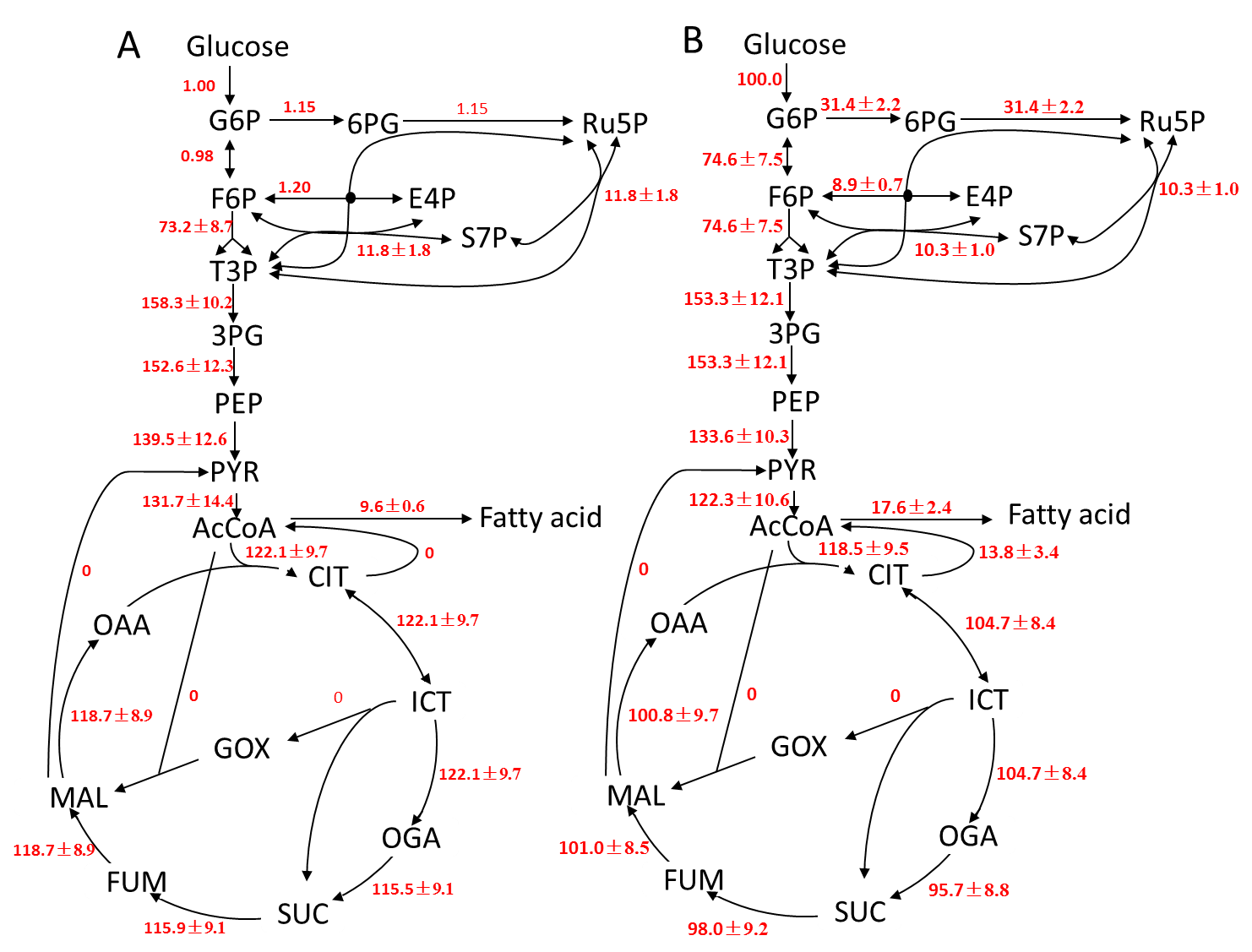

Supplement: S2 Fig — Metabolic flux distribution of Y. lipolytica cultivated in high nitrogen medium (A) and low nitrogen medium (B). A value of 0 in the metabolic flux map indicates a very low flux (below 0.5). (TIF) [file pone.0159187.s002.tif]
