# Supplementary material for: Metabolic Flux Analysis of Lipid Biosynthesis in the Yeast Yarrowia lipolytica Using 13C-Labled Glucose and Gas Chromatography-Mass Spectrometry
Source: PLoS One. 2016 Jul 25;11(7):e0159187. doi: 10.1371/journal.pone.0159187 (PMC4959685; doi:10.1371/journal.pone.0159187)
Supplement: S1 File — (DOCX) [file pone.0159187.s003.docx]

Biochemical reactions for constructing the metabolic networks of *Y. lipolytica* (m, mitochondrial; c, cytoplasmic.)

1. Gluc + ATP -> G6P + ADP

2. F6P + ATP -> FBP + ADP

3. FBP <=> DHAP + GAP

4. DHAP <=> GAP

5. GAP + NAD + ADP + Pi <=> 3PG + ATP + NADH

6. 3PG <=> PEP

7. PEP + ADP -> Pyr + ATP

8. G6P + ATP -> ADP + ADPG

9. G6P + NADP -> 6PG + NADPH

10. ATP + cCit -> ADP + cOAA + AcCoA

11. 6PG + NADP -> Ru5P + CO_2_ + NADPH

12. Ru5P <=> X5P

13. Ru5P <=> R5P

14. X5P + R5P <=> GAP + S7P

15. X5P + E4P <=> GAP + F6P

16. S7P + GAP <=> E4P + F6P

17. mPyr + NAD -> mAcCoA + CO_2_ + NADH

18. mAcCoA + mOAA -> mCit

19. mCit <=> ICit

20. ICit + NADP <=> AKG + CO_2_ + NADPH

21. AKG + NAD -> SucCoA + CO_2_ + NADH

22. SucCoA + ADP + Pi <=> Suc + ATP

23. Suc + FAD <=> Fum + FADH_2_

24. Fum <=> mMal

25. mMal + NAD <=> mOAA + NADH

26. ICit -> Glyox + Suc

27. AcCoA + Glyox -> mMal

28. cMal + NADP -> cPyr + CO_2_ + NADPH

29. PEP + CO_2_ -> cOAA + Pi

30. cOAA + ATP -> PEP + CO_2_ + ADP

31. DHAP + NADH <=> Glyc3P + NAD

32. 8 cAcCoA + 7 ATP + 14 NADPH -> C16:0 + 7 ADP + 14 NADP

33. C16:0 + NADH + O_2_ -> C16:1 + NAD

34. 9 cAcCoA + 8 ATP + 16 NADPH -> C18:0 + 8 ADP + 16 NADP

35. C18:0 + NADH + O_2_ -> C18:1 + NAD

36. C18:1 + NADH + O_2_ -> C18:2 + NAD

37. AKG + NADPH + NH_3_ -> Glu + NADP

38. Glu + ATP + NH_3_ -> Gln + ADP + Pi

39. Glu + 2 NADPH + ATP -> Pro + 2 NADP + ADP + Pi

40. Glu + CO_2_ + Gln + NADPH + Asp + AcCoA + 5 ATP -> Arg + AKG + NADP + Fum + Ac + 5 ADP + 5 Pi

41. OAA + Glu -> Asp + AKG

42. Asp + NH_3_ + 2 ATP -> Asn + 2 ADP + 2 Pi

43. Pyr + Glu -> Ala + AKG

44. 3PG + Glu + NAD -> Ser + NADH + AKG + Pi

45. Ser + THF <=> Gly + MEETHF

46. Gly + THF + NAD <=> CO_2_ + MEETHF + NH_3_ + NADH

47. Thr + NAD -> Gly + AcCoA + NADH

48. Ser + AcCoA + SO_4_ + 3 ATP + 4 NADPH -> Cys + Ac + 4 NADP + 3 ADP + 3 Pi

49. Asp + Pyr + Glu + 2 NADPH + ATP + SucCoA -> LL-DAP + AKG + 2 NADP + ADP + Pi + Suc

50. LL-DAP -> Lys + CO_2_

51. Asp + 2 NADPH + 2 ATP -> Thr + 2 NADP + 2 ADP + 2 Pi

52. Asp + METHF + Cys + 2 NADPH + ATP + SucCoA -> Met + Pyr + 2 NADP + ADP + Pi + Suc + NH_3_ + THF

53. 2 Pyr + NADPH + Glu -> Val + CO_2_ + NADP + AKG

54. 2 Pyr + AcCoA + Glu + NADPH + NAD -> Leu + 2 CO_2_ + AKG + NADP + NADH

55. Thr + Pyr + Glu + NADPH -> Ile + CO_2_ + AKG + NADP + NH_3_

56. E4P + 2 PEP + Glu + NADPH + ATP -> Phe + CO_2_ + AKG + NADP + ADP + 4 Pi

57. E4P + 2 PEP + Glu + NADPH + NAD + ATP -> Tyr + CO_2_ + AKG + NADP + NADH + ADP + 4 Pi

58. E4P + 2 PEP + R5P + Ser + Gln + NADPH + 3 ATP -> Trp + CO_2_ + Pyr + GAP + Glu + NADP + 3 ADP + 6 Pi

59. R5P + FTHF + Gln + Asp + 5 ATP + 2 NAD -> His + 2 NADH + AKG + Fum + 5 ADP + 6 Pi + THF

60. NADH + 0.5 O_2_ + 3 ADP + 3 Pi -> 2.5 ATP + NAD

61. FADH2 + 0.5 O_2_ + 2 ADP + 2 Pi -> 1.5 ATP + FAD

62. NADH + NADP <=> NADPH + NAD

63. 2 ATP + Gln + CO_2_ -> 2 ADP + Glu + CarP

64. R5P + ATP -> PRPP + ADP

65. 4 ATP + CO_2_ + PRPP + Asp + Gly + 2 Gln + 2 FTHF -> Fum + 2 Glu + 4 ADP + IMP + 2 THF

66. 3 ATP + Gln + NAD + IMP -> NADH + Glu + rGTP + 3 ADP

67. 3 ATP + Asp + IMP -> Fum + rATP

68. CarP + Asp + NAD + PRPP + 2 ATP -> 2 ADP + rUTP + CO_2_ + NADH

69. ATP + Gln + rUTP -> rCTP + ADP + Glu

70. NADPH + rCTP -> dCTP + NADP

71. dCTP + METHF -> dTTP + NH_3_ + THF

72. NADPH + rATP -> dATP + NADP

73. NADPH + rGTP -> dGTP + NADP

74. mCIT > cCIT

75. cOAA + NADH <=> cMal + NAD

76. cMAL > mMAL

77. mMAL +NAD > mPYR +NADH+ CO_2_

78. mPYR + ATP + CO_2_ > mOAA + ADP + Pi

79. cPYR > ACA + CO_2_

80. ACA + NADP = ACE + NADPH

81. ACE + 2ATP > cAcCoA + 2 ADP + 2Pi

**Abbreviations**

**G6P** glucose-6-phosphate

**P5P** pentose-5-phosphate

**F6P** fructose-6-phosphate

**6PG** 6- phosphate-gluconate

**T3P** triose-3**-** phosphate

**PYR** pyruvate

**S7P** sedoheptulose-7-phosphate

**E4P** erythrose-4-phosphate

**PGA** phosphoglycerate

**PEP** phosphoenolpyruvate

**AcCoA** acetyl coenzyme A

**ICT** isocitrate

**OGA** α-ketoglutarate

**OAA** oxaloacetate

**FUM** fumarate

**MAL** malate
