# Supplementary material for: Metabolic Flux Analysis of Lipid Biosynthesis in the Yeast Yarrowia lipolytica Using 13C-Labled Glucose and Gas Chromatography-Mass Spectrometry
Source: PLoS One. 2016 Jul 25;11(7):e0159187. doi: 10.1371/journal.pone.0159187 (PMC4959685; doi:10.1371/journal.pone.0159187)
Supplement: S1 Table — The theoretical FL value is 0.2, equal to the labeling content of the input glucose. (DOCX) [file pone.0159187.s004.docx]

| Fragments | H-N | L-N |
| --- | --- | --- |
| M_ala_057 | 0.22 | 0.22 |
| M_ala_085 | 0.23 | 0.23 |
| M_asx_057 | 0.21 | 0.20 |
| M_asx_085 | 0.19 | 0.21 |
| M_asx_302 | 0.22 | 0.19 |
| M_glx_057 | 0.20 | 0.20 |
| M_glx_085 | 0.21 | 0.20 |
| M_glx_302 | 0.16 | 0.20 |
| M_gly_057 | 0.24 | 0.24 |
| M_gly_085 | 0.23 | 0.25 |
| M_ile_015 | 0.20 | 0.19 |
| M_ile_085 | 0.21 | 0.20 |
| M_leu_015 | 0.21 | 0.18 |
| M_leu_085 | 0.20 | 0.19 |
| M_phe_057 | 0.20 | 0.20 |
| M_phe_085 | 0.22 | 0.21 |
| M_phe_302 | 0.21 | 0.20 |
| M_phe_sc | 0.24 | 0.23 |
| M_pro_057 | 0.22 | 0.19 |
| M_pro_085 | 0.21 | 0.20 |
| M_ser_057 | 0.21 | 0.20 |
| M_ser_085 | 0.24 | 0.20 |
| M_ser_302 | 0.24 | 0.23 |
| M_thr_057 | 0.20 | 0.20 |
| M_thr_085 | 0.19 | 0.21 |
| M_thr_sc | 0.21 | 0.23 |
| M_val_057 | 0.22 | 0.21 |
| M_val_085 | 0.22 | 0.21 |
| M_val_302 | 0.25 | 0.24 |
| M_tyr_057 | 0.21 | 0.21 |
| M_tyr_085 | 0.21 | 0.21 |
| M_tyr_302 | 0.20 | 0.19 |
